# Identification of immune-associated genes for the diagnosis of ulcerative colitis-associated carcinogenesis via integrated bioinformatics analysis

**DOI:** 10.3389/fonc.2024.1475189

**Published:** 2024-11-08

**Authors:** Xueyu Cang, Ning Li, Jihan Qi, Hongliang Chen, Hui Xing, Jiawei Qiu, Yingying Tian, Shiling Huang, Pengchao Deng, Feiyang Gao, Ram Prasad Chaulagain, Ubaid Ullah, Chunjing Wang, Lina Liu, Shizhu Jin

**Affiliations:** ^1^ Department of Gastroenterology and Hepatology, The Second Affiliated Hospital of Harbin Medical University, Harbin, China; ^2^ Department of General Surgery, The Second Affiliated Hospital of Harbin Medical University, Harbin, China; ^3^ Department of Endoscopic Center, The Second Affiliated Hospital of Harbin Medical University, Harbin, China

**Keywords:** ulcerative colitis, ulcerative colitis-associated carcinogenesis, bioinformatics analysis, immune infiltration, diagnosis biomarker

## Abstract

**Background:**

UC patients suffer more from colorectal cancer (CRC) than the general population, which increases with disease duration. Early colonoscopy is difficult because ulcerative colitis-associated colorectal cancer (UCAC) lesions are flat and multifocal. Our study aimed to identify promising UCAC biomarkers that are complementary endoscopy strategies in the early stages.

**Methods:**

The datasets may be accessed from the Gene Expression Omnibus and The Cancer Genome Atlas databases. The co-expressed modules of UC and CRC were determined via weighted co-expression network analysis (WGCNA). The biological mechanisms of the shared genes were exported for analysis using the Gene Ontology and Kyoto Encyclopedia of Genes and Genomes. To identify protein interactions and hub genes, a protein-protein interaction network and CytoHubba analysis were conducted. To evaluate gene expression, external datasets and experimental validation of human colon tissues were utilized. The diagnostic value of core genes was examined through receiver operating characteristic (ROC) curves. Immune infiltration analysis was employed to investigate the associations between immune cell populations and hub genes.

**Results:**

Three crucial modules were identified from the WGCNA of UC and CRC tissues, and 33 coexpressed genes that were predominantly enriched in the NF-κB pathway were identified. Two biomarkers (CXCL1 and BCL6) were identified via Cytoscape and validated in external datasets and human colon tissues. CRC patients expressed CXCL1 at the highest level, whereas UC and CRC patients showed higher levels than the controls. The UC cohort expressed BCL6 at the highest level, whereas the UC and CRC cohorts expressed it more highly than the controls. The hub genes exhibited significant diagnostic potential (ROC curve > 0.7). The immune infiltration results revealed a correlation among the hub genes and macrophages, neutrophils and B cells.

**Conclusions:**

The findings of our research suggest that BCL6 and CXCL1 could serve as effective biomarkers for UCAC surveillance. Additionally, they demonstrated a robust correlation with immune cell populations within the CRC tumour microenvironment (TME). Our findings provide a valuable insight about diagnosis and therapy of UCAC.

## Introduction

Ulcerative colitis (UC), known as inflammatory bowel disorder, is characterized by mucopurulent and bloody stools and relapsing-remitting diarrhea (1–3). Studies have consistently shown a correlation between UC and the risk of colorectal cancer (CRC) (4). Chronic inflammation in the intestinal tract generates proinflammatory mediators and causes cell proliferation, which influences the immune system and increases cancer risk in UC patients, according to growing evidence (5). In the case of intestinal barrier impairment, exposure to luminal antigens can lead to excessive infiltration of immune-related cells and heightened production of chemokines in the intestinal lamina propria (5–7). The infiltration cell population comprises dendritic cells, neutrophils, and macrophages. Activated immune cells interact with each other through direct or indirect contact by releasing various cytokines (6). The complex intestinal microenvironment complicates the exploration of pathogenesis behind UCAC (8, 9). Meanwhile, the specific genes responsible for regulating UC progression and the extent of their expression alterations during colitis-associated malignant transformation remain unclear. Although colonoscopic detection has served as the standard strategy for screening for malignancies in UC patients for a long time, early diagnosis remains tricky due to the flat appearance and presence of multifocal lesions (10, 11). It is therefore necessary to ascertain the potential mechanisms that may led to the development of UC-CRC and to investigate promising biomarkers as a means of facilitating early diagnosis.

Studies on the progression from UC to CRC over the past few decades have examined a variety of factors, including the length of the disease, its stages, the extent of the lesion, the age at onset, genders, and whether or not primary sclerosing cholangitis (PSC) is present (12, 13). However, a variety of factors affected tumorigenesis; these studies only focused on the disease itself or the comparison between the tumor cohort and the controls. The application of bioinformatics methods has provided unparalleled insight into disease mechanisms and biomarker identification in recent years (14). A variety of studies have been carried out in the detection of UC-CRC fields, such as biomarkers from blood, fecal, and miRNA, as well as the alteration of intestinal flora et al. (15–19). However, there are also some deficiencies, including poor specificity, detection complexity, lower acceptance of patients, and high cost.

In our research, using database mining, we analyzed multiple microarray datasets correlated with UC and CRC. We identified gene modules related to the UC-CRC via weighted gene coexpression network analysis (WGCNA) and Venn diagram analysis. Next, we applied enrichment analyses to the module genes, revealing important associated biological processes. Cytoscape identified two key genes, CXCL1 and BCL6. The immune correlation analysis of key genes reveals a strong relationship between these genes and immune cells (macrophages, neutrophils, and B cells) involved in colitis-associated cancer. Furthermore, we verified the presence of CXCL1 and BCL6 in external datasets and human colon tissues. Instead of concentrating solely on exploring the relationship between healthy and diseased groups or a single immune cell, our research utilized bioinformatics techniques to extract and analyze transcriptome data. This approach yielded disease-related biomarkers that exhibit a strong relation with immune cell populations in the TME.

## Materials and methods

### Data sources

**Figure 1:**
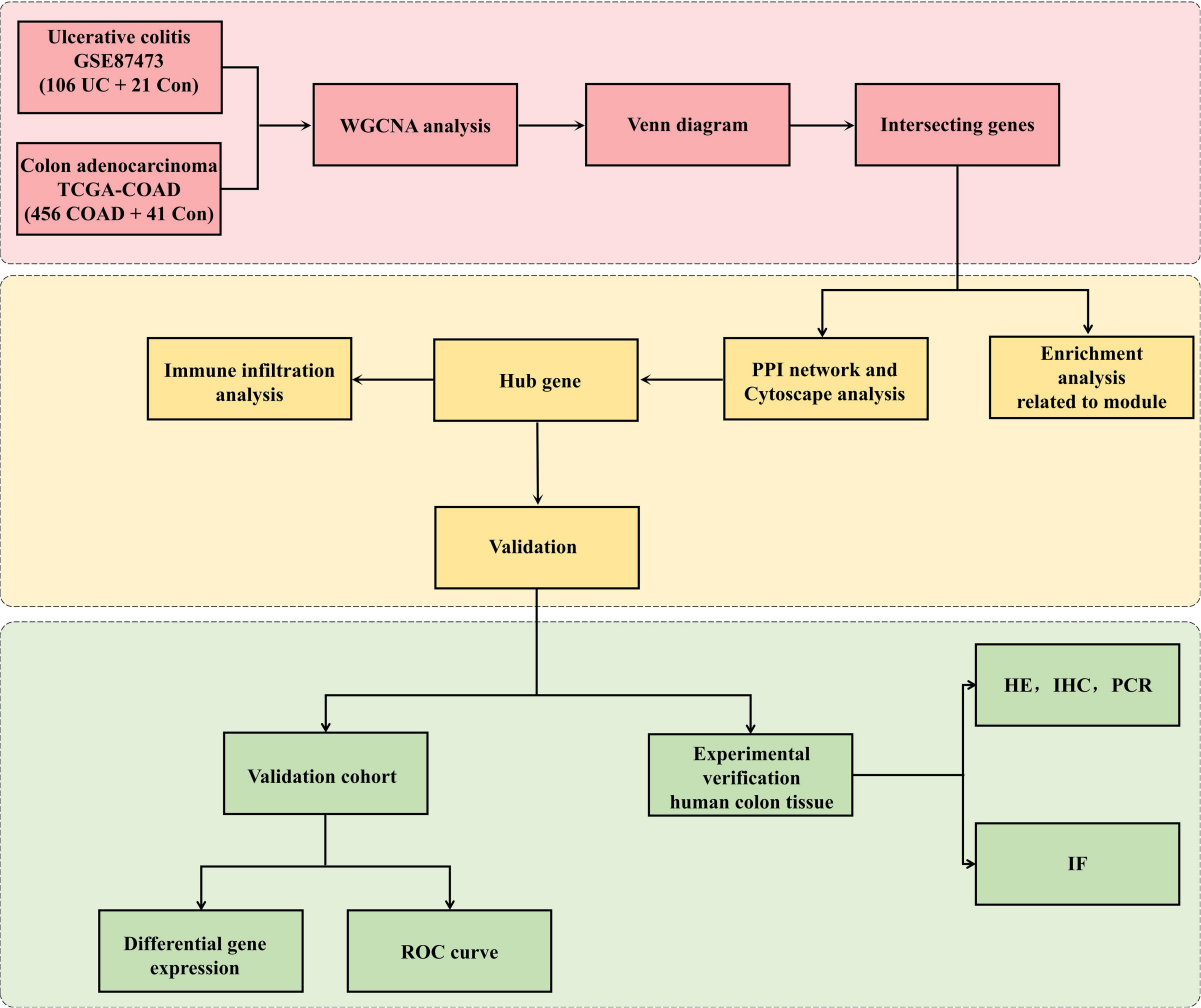
Research design flowchart. The RNA sequences of colon tissues from UC, CRC cohorts and the controls were obtained from the GEO and TCGA databases. The datasets were pretreated via R software. WGCNA was used to identify the significant coexpression modules between UC and CRC. Then, the coexpressed genes were visualized with a Venn diagram. GO and KEGG analyses were applied to coexpressed genes. The top 5 hub genes related to UC-CRC were screened via cytoHubba. The expression of the five hub genes was verified via validation cohorts, and the hub genes was estimated via ROC curve and immune infiltration analyses. Finally, experimental verification (HE, WB, IHC and IF analysis) of human colon tissues was applied to affirm the differential expression of hub genes among UC patients, CRC patients and controls.

**Figure 1** exhibitied the workflow of our study. Microarray datasets of UC came from the GEO database (http://www.ncbi.nlm.nih.gov/geo), and GEO include numerous high-throughput datasets and expression microarray datasets. We searched related gene expression profiles and UC patients information with the key words “colon mucosal” and “ulcerative colitis” in the GEO database. We got the transcriptome data, patients information and healthy control data from the TCGA database. First, gene expression profiling should include case and control groups. Second, to maintain the accuracy of WGCNA, more than 10 samples were included in each dataset. Third, all the samples were colon tissues. Fourth, these gene matrices must provide raw data for further analysis. Finally, TCGA-COAD in the TCGA dataset and GSE87473, GSE87466, GSE39582, GSE92415 and GSE20916 in the GEO dataset were chosen for subsequent research. The probe expression matrix was converted into a gene expression matrix based on platform annotation files.

### Co-expression module identification

WGCNA explored gene module associations with disease traits by analyzing coexpressed gene modules with high biological significance (20). Hence, modules associated with UC and CRC were detected in the GSE87473 and TCGA-COAD datasets by using “WGCNA package”. In our study, we selected the first 10,000 genes with large variation based on their variance for further analysis. To omit unqualified genes and samples, Hclust (hierarchical clustering) analysis was performed in R prior to analysis. First, based on the criterion of R^2^ > 0.85, a candidate soft-thresholding power β (ranging from 1-20) was computed for the scale-free topology using the pickSoftThreshold function. The subsequent step involved the construction of an adjacency matrix utilizing the soft power value and gene correlation matrix derived from Pearson analysis. This adjacency matrix was then transformed into a topological overlap and corresponding dissimilarity matrix. Subsequently, coexpressed gene modules were determined via hierarchical clustering, and subsequently, we acquired a dynamic tree. The minimum number of modules was 30. Finally, to determine each module’s expression profile, we calculated the module eigengene and its association with clinical features. Other parameters were as follows: mergeCutHeight = 0.2, deepSplit = 2 and networkType = “unsigned”.

### Functional enrichment analysis

For acquiring the potential inner mechanisms and functions of coexpressed genes, Gene Ontology (GO) annotation and Kyoto Encyclopedia of Genes and Genomes (KEGG) analysis were performed with the “ClusterProfiler package”. GO included biological process (BP), cellular component (CC) and molecular function (MF) (21). KEGG is a comprehensive database containing valuable information on gene pathways (22). Furthermore, barplots and circos plots were employed to display the outcome of enrichment analysis.

### Construction of the PPI network

The online search tool STRING (http://string-db.org) (version 12.0) was applied to search for interrelationship among protein-coding genes of interest (minimum required interaction score: 0.15). PPI network could be visualized via the utilization of Cytoscape (version 3.9.1) (23). As a plugin, MCODE was applied to filter critical functional interaction genes, with K-core=2, degree cut-off=2, max depth=100 and node score cut-off=0.2 (24). CytoHubba was utilized to figure out the hub genes. For the purpose of evaluating and selecting hub genes, five common algorithms (MNC, DMNC, degree, MCC and EPC) were applied. The subsequent step involved the utilization of GeneMANIA (http://www.genemania.org/), which is used for internal association detection within gene sets and for establishing coexpression networks for central genes (25).

### Expression verification of hub genes

Expression validation of these genes in GSE87466 and GSE39582 datasets, respectively. The GSE87466 dataset consisted of 21 normal people and 87 ulcerative colitis patients. GSE39582 is composed of 19 normal people and 566 CRC patients. The GSE87466 and GSE39582 datasets were obtained from human intestinal tract tissue. We used the t test to compare the two datasets.

### Receiver operating characteristic curves

With the “pROC” package, Receiver Operating Characteristic (ROC) curves were established in order to estimate the diagnosis capacity of key genes (26). The AUC values and corresponding 95% CIs were calculated, with an AUC > 0.7 indicating high diagnostic value.

### Analysis of immune infiltration

To evaluate disease immune microenvironment and immune cell infiltration, the “cibersort” package was utilized (27). The distribution of immune cells across different samples was visually represented using a bar plot. Additionally, a correlation heatmap depicting the interrelationships among 22 immune cell types was generated utilizing the “corrplot” package. Vioplotting was conducted to compare the proportions of diverse immune cells in the UC and healthy cohorts. Finally, Spearman’s correlation was performed to reveal the relationships between hub biomarker expression and infiltrating immune cells.

### Verification of the human colonic mucosa

#### Collection of clinical samples

In this study, clinical partial intestinal biopsy tissue samples, including adjacent cancerous tissue from colorectal cancer patients, colon tissue from UC patients, and cancer tissue from colorectal cancer patients, were available from The Second Affiliated Hospital of Harbin Medical University. All patients suffered from UC and CRC after endoscopic examination and pathological diagnosis. The remaining colon tissue from UC and CRC patients was collected as our validation cohort. The protocols were approved via the Ethics Committee of the Second Affiliated Hospital, Harbin Medical University (KY2023-051).

### Hematoxylin-eosin staining analysis

The fresh colon tissues were fixed in 4% paraformaldehyde solution overnight, dehydrated and embedded in paraffin. Subsequently, paraffin section were cut into 5-μm sections and immersed in a hematoxylin-eosin blend for uniform staining. Using an microscope (Olympus, Japan), we observed and photographed colon tissue slices.

### Immunohistochemical analysis

After fixation in 4% paraformaldehyde for 12 hours, *P*araffin sections of the colon tissues were prepared. A thermostat set at 80°C was applied to heat the sections overnight. Afterwards, the sections were dewaxed in xylene and dehydrated in graded ethanol, followed by subsequent inactivation of endogenous peroxidase activity using a 3% H2O2 solution and then antigen retrieval with ethylenediaminetetraacetic acid. Subsequently, they were blocked with 5% goat serum (ABS933, Absin, Shanghai, China) for 30 minutes and incubated overnight at 4°C with *P*rimary antibodies (anti-CXCL1[1:100, 12335-1-AP, *P*roteintech] and anti-BCL6 [1:1000, 66340-1-Ig, Proteintech]). The following day, the sections were incubated at 37°C without light for 1 hour with secondary antibodies (goat anti-rabbit [RGAR011, *P*roteintech] and anti-mouse [RGAM011, Proteintech]). After washing again with *P*BS, the chromogenic substrate diaminobenzidine (ZLI-9017, ZSGB-BIO, China) was applied to the sections. Staining was finished when positive cells exhibited deep staining while maintaining an unstained or lightly stained background under a microscope (Olympus, Japan). The paraffin sections were subsequently counterstained with hematoxylin followed by thorough rinsing in water. Dehydration was achieved using gradient alcohol and xylene before the samples were sealed appropriately in resin. Finally, the dried sections were photographed for analysis by ImageJ software and GraphPad Prism 8 software.

### Western blot analysis

In liquid nitrogen, samples of colon tissues were thoroughly ground and in lysis buffer, contain protease inhibitors, samples of colon tissues were homogenized. The tissues were placed in a centrifuge tube and centrifuged at 12,000 rpm for 20 min at 4°C. The BCA approach was applied to measure the total protein concentration in the supernatant. We heated and denatured the protein solution at 100°C for 5 minutes. After removing solution, placed them on ice and stored at a temperature of 20°C. We further transferred the protein samples to a polyvinylidene fluoride (PVDF) membranes. The PVDF membrane was blocked with 5% skim milk. The membrane was washed twice with TBST for 5 minutes each and subsequently incubated overnight at 4°C with primary antibodies against CXCL1 (1:500, 12335-1-AP, Proteintech), BCL6 (1:1000, 66340-1-Ig, Proteintech), and β-actin (1:1000, 7A-09, ab8226, ZSGB-BIO). On the following day, the PVDF membrane was exposed to the secondary antibodies (goat anti-rabbit [RGAR001, Proteintech] and anti-mouse [RGAM001, Proteintech]) for 1 hour at 37°C. Subsequently, the protein bands were immersed in enhanced chemiluminescence solution, and images were captured using a gel imaging system.

### Immunofluorescence staining

After the colon tissues were rinsed with saline solution, they were gently dried using filter paper to eliminate any residual moisture. The tissue blocks were then carefully sectioned into 1 cm3 pieces and embedded in optimal cutting temperature compound (OCT). Frozen colon tissues were sliced into 5-μm sections, followed by treatment with 0.1% Triton X-100 to enhance cell permeability. After blocking with 5% goat serum (ABS933, Absin, China) for 30 minutes at a temperature of 37°C, primary antibodies, including anti-CXCL1 (1:100, 12335-1-AP, Proteintech) and anti-BCL6 (1:1000, 66340-1-Ig, Proteintech), were added and incubated overnight at 4°C. On the following day, goat anti-rabbit secondary antibody (RGAR002; Proteintech, China) and anti-mouse secondary antibody (RGAM002; Proteintech, China) were applied to the colon sections, which were then incubated for one hour at a temperature of 37°C in the dark. Nuclei staining was performed using DAPI (Beyotime, China) for 5 min before images were captured under a laser confocal microscope (Zeiss, Germany). The results were semiquantitatively analyzed with GraphPad Prism 8.2.1 and Image J software.

### Statistical analysis

The statistical analysis and image preparation were completed via R studio (version 4.1.3) and GraphPad Prism 8.2.1, respectively. Continuous variables with a normal distribution are described as the mean ± standard deviation (SD). One-way analysis of variance was employed for data analysis involving more than two groups. Figures were edited using Adobe Illustrator (AI), PhotoShop (PS), and Image J. Statistical significance was defined when *P* < 0.05.

## Results

### Dataset information

Five GEO datasets and one TCGA dataset, namely, GSE87473, TCGA-COAD, GSE87466, GSE92415, GSE39582 and GSE20916, were selected. Information on each dataset is shown in **Table 1**. We further coupled the GSE87473 and TCGA-COAD cohorts as training sets for WGCNA and the other cohorts as verification sets to validate the expression of hub genes.

**Table 1:** Dataset information used in this study.

ID	Dataset number	Platform	Sample	Diseases	Group
1	GSE87473	GPL13158	106 patients and 21 controls	UC	Discovery cohort
2	TCGA-COAD	TCGA	456 patients and 41 controls	CRC	Discovery cohort
3	GSE87466	GPL13158	87 patients and 21 controls	UC	Validation cohort
4	GSE39582	GPL570	566 patients and 19 controls	CRC	Validation cohort
5	GSE92415	GPL13158	87 patients and 21 controls	UC	Validation cohort
6	GSE20916	GPL570	66 patients and 34 controls	CRC	Validation cohort

### WGCNA and key module gene identification

**Figure 2:**
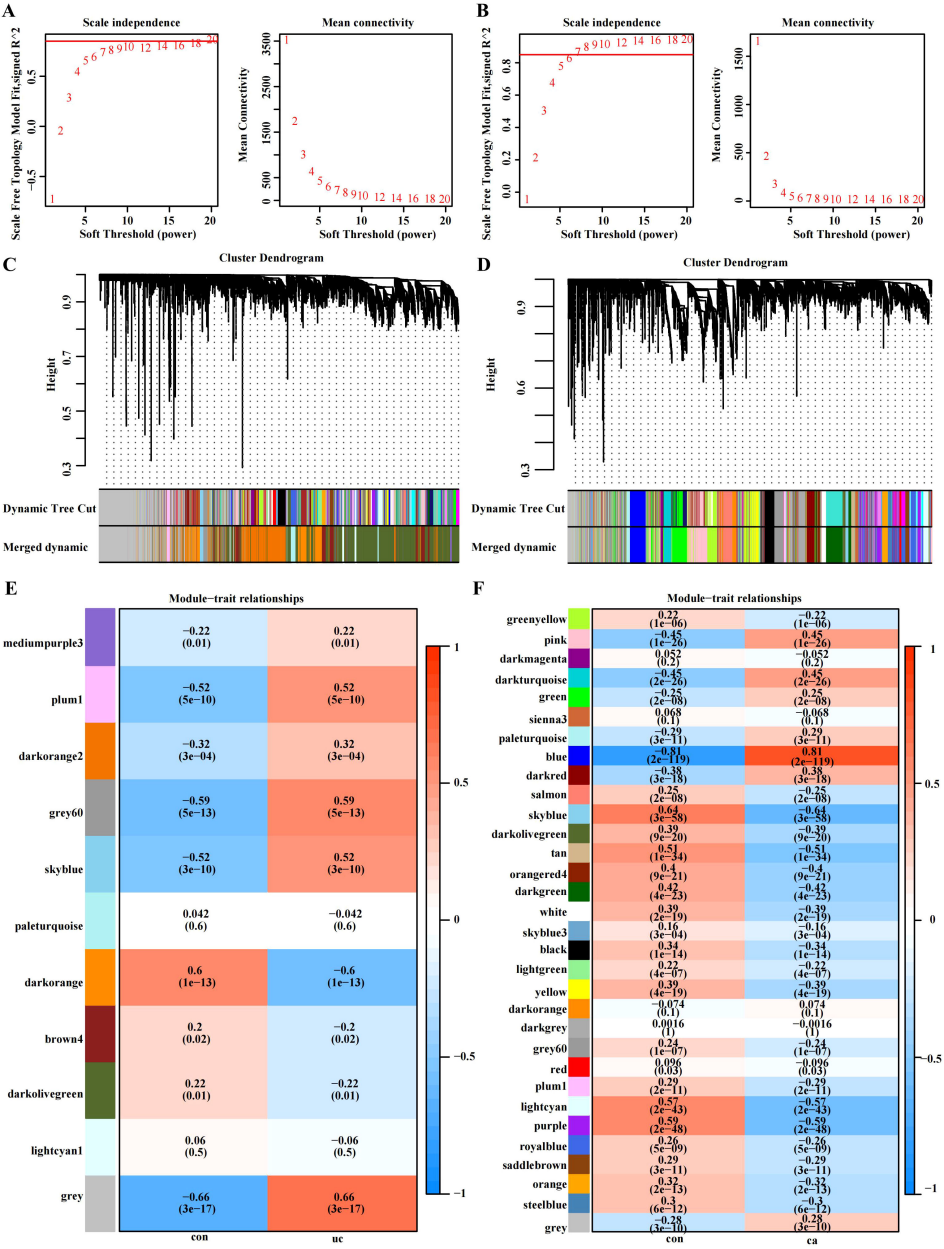
WGCNA of the UC group (GSE87473) and CRC group (TCGA-COAD). **(A)** β= 20 is the soft threshold in UC patients according to the combined analysis of scale independence and average connectivity. **(B)** β = 7 was selected as the soft threshold in CRC. **(C, D)** Gene dendrograms were obtained by hierarchical clustering. The different colored rows under the dendrogram show the gene coexpression module assignments determined by the dynamic tree cut method. **(E, F)** Relationships between modules and traits visualized as a heatmap. Correlations and p values are displayed in each cell. Each row corresponds to the gene module, and each column is related to a clinical trait.

**Figure 3:**
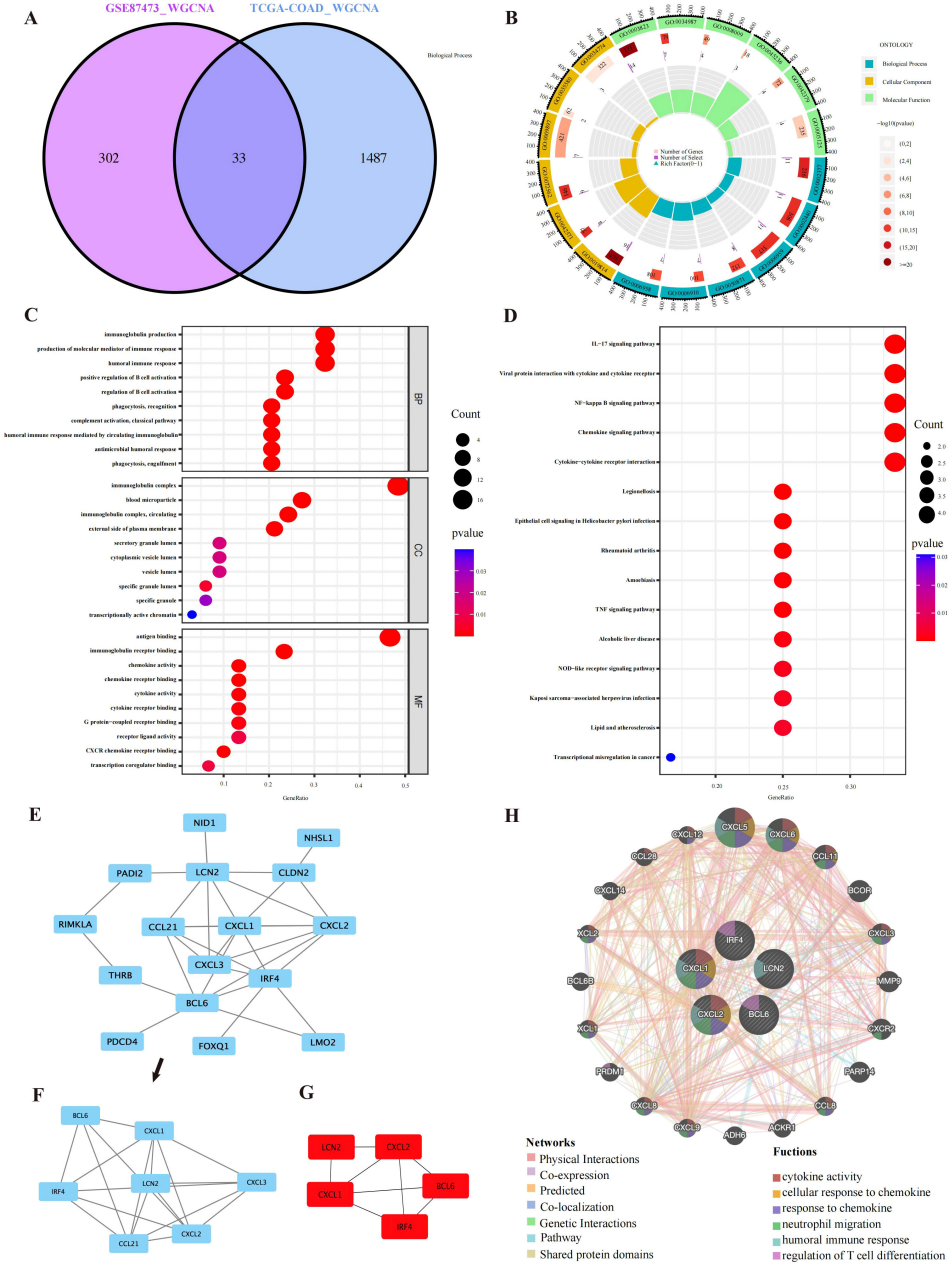
PPI network and enrichment analysis results. **(A)** A total of 33 overlapping genes were identified from the gene intersections in UC and CRC patients via WGCNA. **(B)** GO circle representing the GO enrichment analysis of the overlapping genes. **(C)** GO functional enrichment analysis of the overlapping genes, comprising BP, CC, and MF. The different GO terms are displayed on the y-axis. Gene ratios enriched in terms are shown on the x-axis. **(D)** The 15 most significantly enriched KEGG pathways. **(E, F)** Sixteen interacting genes and important modules visualized via MCODE. **(G)** Identification of the top five key genes by multiple MCC, DMNC, MNC, Degree and EPC methods. **(H)** GeneMANIA was applied to explore internal association of overlapping genes and their coexpressed genes.

We identified 11 functional modules in GSE87473 by WGCNA between the UC and control groups. We chose an optimal β value = 20 (scale-free R^2^ = 0.85) as the soft threshold according to the scale-free topology criterion and average connectivity (**Figure 2A**). The Spearman correlation coefficient was used to create a heatmap that illustrates the relationships between modules and traits. This allowed for an evaluation of the associations between the modules and the disease (**Figures 2C, E**). Three modules, namely, ‘skyblue’, ‘plum1’, and ‘gray60’, showed significantly positive correlations with UC and were considered UC-associated modules. (sky blue module: r= 0.52, p = 3e−10, genes= 116; plum1: r=0.52, p= 5e−10, genes= 90; grey60: r= 0.59, p = 5e−13, genes=169). By setting the soft threshold to 7 (**Figure 2B**), 32 modules were identified in TCGA-COAD, with the modules “blue” (r = 0.81, p = 2e−119, genes = 480), “pink” (r = 0.45, p = 1e−26, genes = 834) and “darkturquoise” (r=0.45, p = 2e−26, genes = 206) being significantly correlated with CRC (**Figures 2D, F**). A Venn diagram was constructed, and 33 intersecting genes were obtained (**Figure 3A**).

### Functional analysis of co-expressed genes

GO and KEGG analysis were applied to explore the underlying information and pathways associated with coexpressed genes. The genes were predominantly enriched in humoral immune response, molecular mediators of immune response production and biological processes related to immunoglobulin production. In CC, the genes were predominantly enriched in immunoglobulin complexes, immunoglobulin complexes and blood microparticles. Molecular function analysis demonstrated significant enrichment in antigen binding and immunoglobulin receptor binding (**Figures 3B, C**). Furthermore, the coexpressed genes were mainly involved in the NF−kappa B pathway, chemokine signaling pathway, TNF signaling pathway, and cytokine–cytokine receptor interaction (**Figure 3D**). **Supplementary Tables S1**, **S2** show the GO and KEGG analyses. These findings strongly suggest the relationship of proinflammatory factors and disease development.

### PPI network construction

We constructed a network of interactions among 33 overlapping gene proteins, with combined scores exceeding 0.15 (**Supplementary Figure S1**). Eighteen nodes and twenty-six edges in the interaction network were visualized with Cytoscape (**Figure 3E**). The MCODE was applied to find subnetworks and gene cluster modules (scores: 4.0, 5 nodes and 10 edges) (**Figure 3F**).

### Selection and analysis of hub genes

We identified the top five crucial genes (CXCL1, BCL6, CXCL2, LCN2, and IRF4) from the PPI network (**Figure 3G**). Topological analysis algorithms (MCC, DMNC, EPC, MNC and Degree) were applied to comprehensively estimate and identify crucial genes in PPI networks. Detailed information on these genes is provided in **Supplementary Table S3** (**Supplementary Table S3**). Using the GeneMANIA database, we analyzed the coexpression networks and functions of these genes. Our findings revealed a complex protein-protein interaction (PPI) network comprising physical interactions (77.64%), coexpression patterns (8.01%), predicted associations (5.37%), colocalization relationships (3.63%), genetic interactions (2.87%), and pathway connections (1.88%), as depicted in **Supplementary Figure S2**. The predicted genes could be found in the outer ring, whereas core genes were located in the inner ring. In **Figure 3H**, these genes were enriched in neutrophil migration, humoral immune response, cellular response to chemokines, regulation of T-cell differentiation, response to chemokines and cytokine activity.

### Expression verification of hub genes

**Figure 4:**
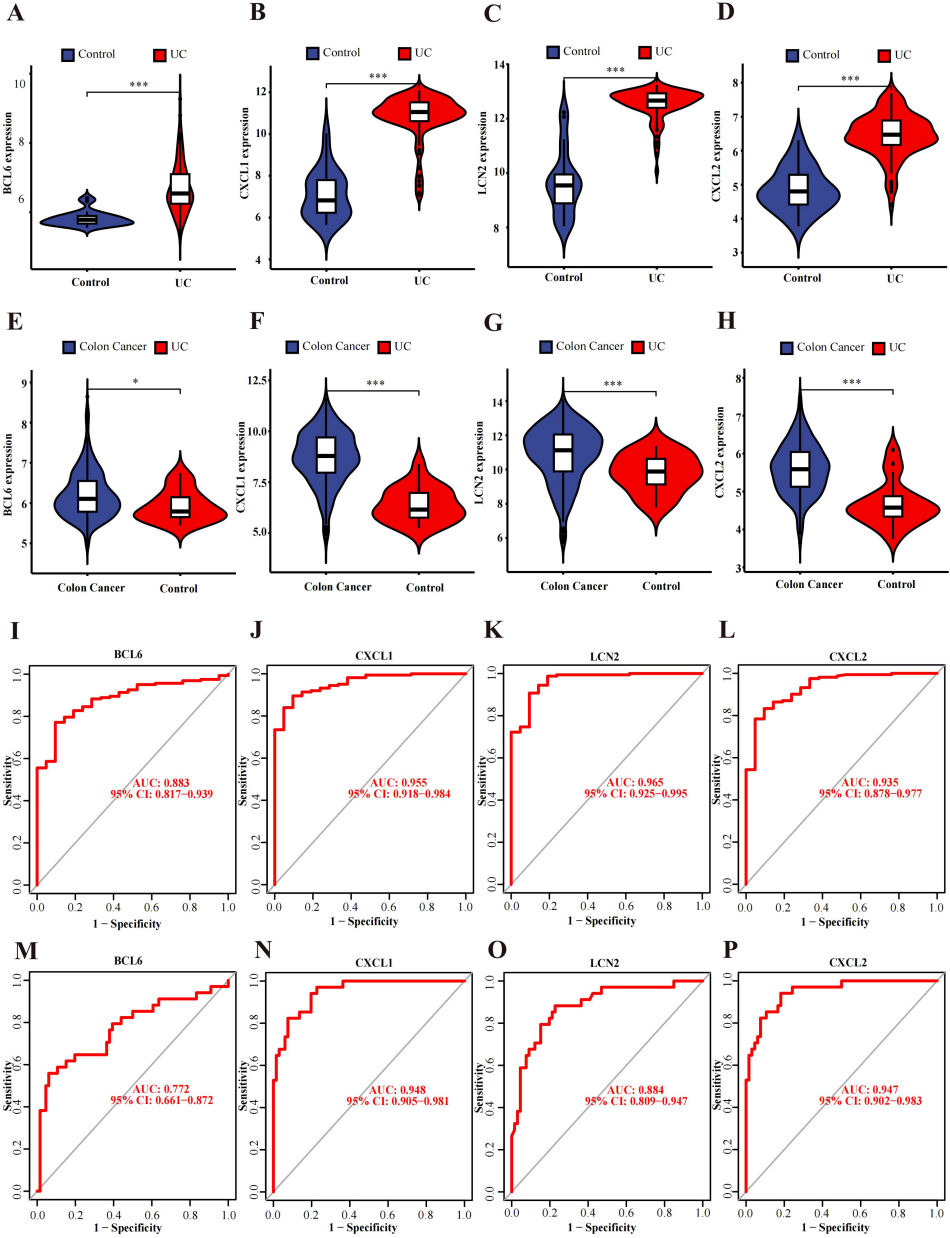
Verification and ROC curves of the hub genes. **(A–D)** The expression verification in the GSE87466 dataset. **(E–H)** Hub gene expression in the GSE39582 dataset. **(I–L)** ROC curves of BCL6, CXCL1, LCN2 and CXCL2 in the GSE92415 dataset. **(M–P)** ROC curves of BCL6, CXCL1, LCN2 and CXCL2 in the GSE20916 dataset. *p<0.05, ***p<0.001.

Differential expression verification was performed in the validation cohort GSE87466 for UC and GSE39582 for CRC. We found that all hub genes (CXCL1, BCL6, CXCL2, and LCN2) showed predominant up-regulation in the UC and CAC patient cohorts in contrast with the controls (**Figures 4A–H**).

### Diagnostic value evaluation of hub genes

ROC curve analysis of the GSE92415 dataset revealed BCL6 (AUC: 0.883, CI: 0.817−0.939), CXCL1 (AUC: 0.955, CI: 0.918−0.984), CXCL2 (AUC: 0.935, CI: 0.878−0.977) and LCN2 (AUC: 0.965, CI: 0.925−0.995) (**Figures 4I–L**). Furthermore, GSE20916 was used to confirm the diagnostic ability of the hub genes BCL6 (AUC: 0.772, CI: 0.661−0.872), CXCL1 (AUC: 0.948, CI: 0.905−0.981), CXCL2 (AUC: 0.947, CI: 0.902−0.983) and LCN2 (AUC: 0.884, CI: 0.809−0.947) (**Figures 4M–P**). The AUC values of four of the five hub genes exceeded 0.85, which indicates that these genes have high diagnostic value.

### Correlations of hub genes and immune cell infiltration

**Figure 5:**
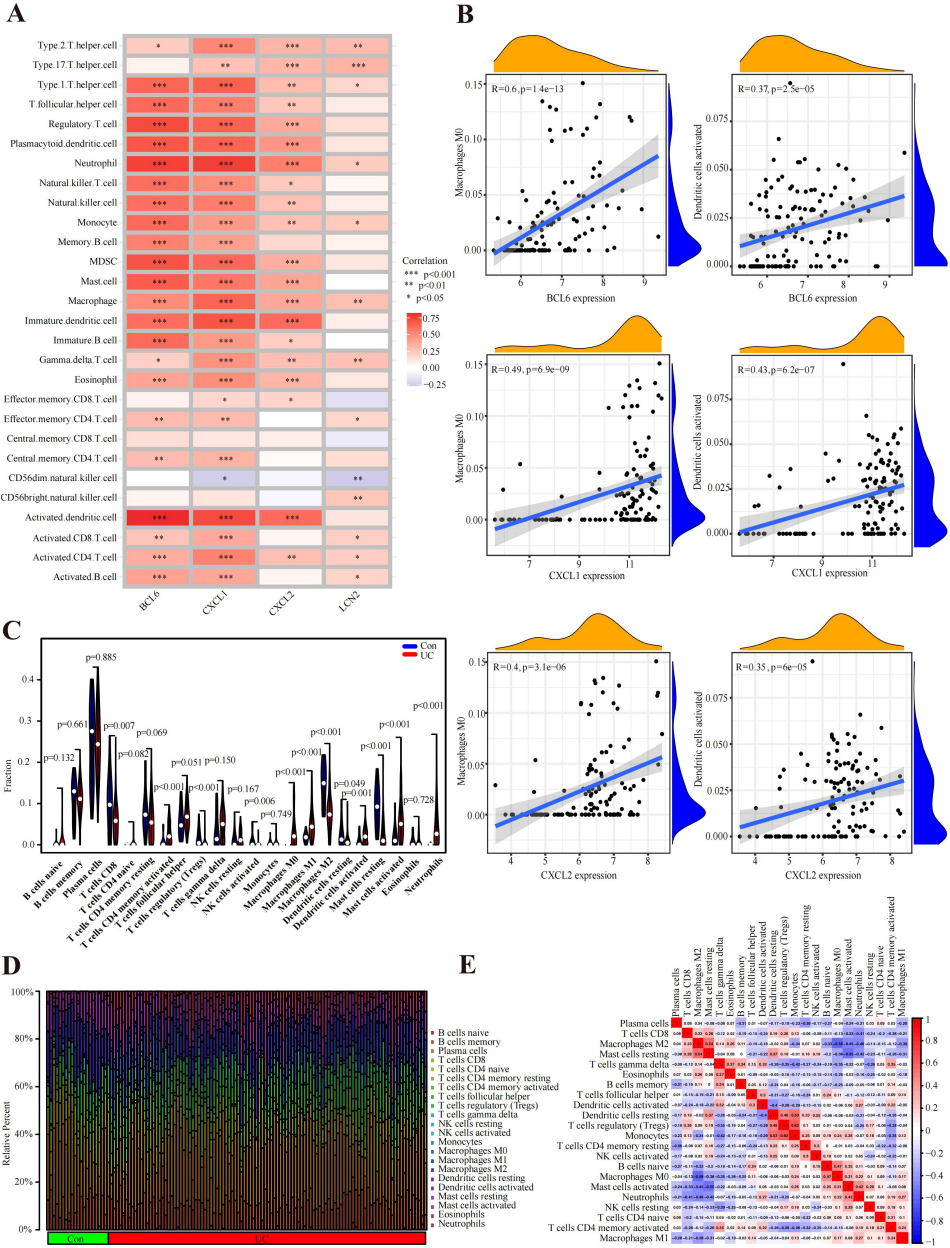
Immune cell infiltration analysis. **(A)** According to the Spearman correlation analysis, the hub genes were strongly correlated with immune cells. **(B)** Correlation results of hub genes and immune cells. **(C)** Violin plot of the proportion of immune cells infiltrating the UC cohort compared to that in the control cohort. **(D)** Proportions of 22 immune cells visualized from the bar plot. **(E)** Heatmap of correlations of different immune cells in UC samples. Red: positive correlation; blue: negative correlation.

In UC samples of GSE87473, macrophage infiltration was strongly correlated with CXCL1, BCL6 and CXCL2. In addition, CXCL1, BCL6 and CXCL2 were associated with the expression of activated dendritic cells and T regulatory cells (**Figure 5A**). Specifically, these genes were strongly related to M0 macrophages (BCL6, R=0.598450467, p=1.36E-13; CXCL1, R=0.487848406, p=6.87E-09; and CXCL2, R=0.402050537, p=3.06E-06) (**Figure 5B**). Moreover, all genes were positively linked to activated dendritic cells (BCL6, R=0.365708474, p=2.54E-05; CXCL1, R=0.426961846, p=6.17E-07; and CXCL2, R=0.349592704, p=6.01E-05) (**Figure 5B**). As shown in **Figure 5D**, the bar plot shows 22 different immune cell type proportions of each sample from the UC and control groups (**Figure 5D**). Violin plots revealed that the UC group had greater numbers of neutrophils, M0 macrophages, M1 macrophages, activated memory CD4^+^ T cells and activated mast cells and lower numbers of M2 macrophages, resting mast cells, CD8^+^ T cells and resting dendritic cells (**Figure 5C**). In addition, resting mast cells were positively related to M2 macrophages (r=0.54), and Monocytes were positively related to resting dendritic cells (r=0.53) and Regulatory T Cells (r=0.62) (**Figure 5E**). Moreover, M0 macrophages were negatively correlated with M2 macrophages (=-0.58), and activated mast cells were negatively linked to resting mast cells (r=-0.55) (**Figure 5E**).

### Verification of the expression of hub genes in human colonic mucosa

#### Endoscopic diagnosis and histological evaluation

**Figure 6:**
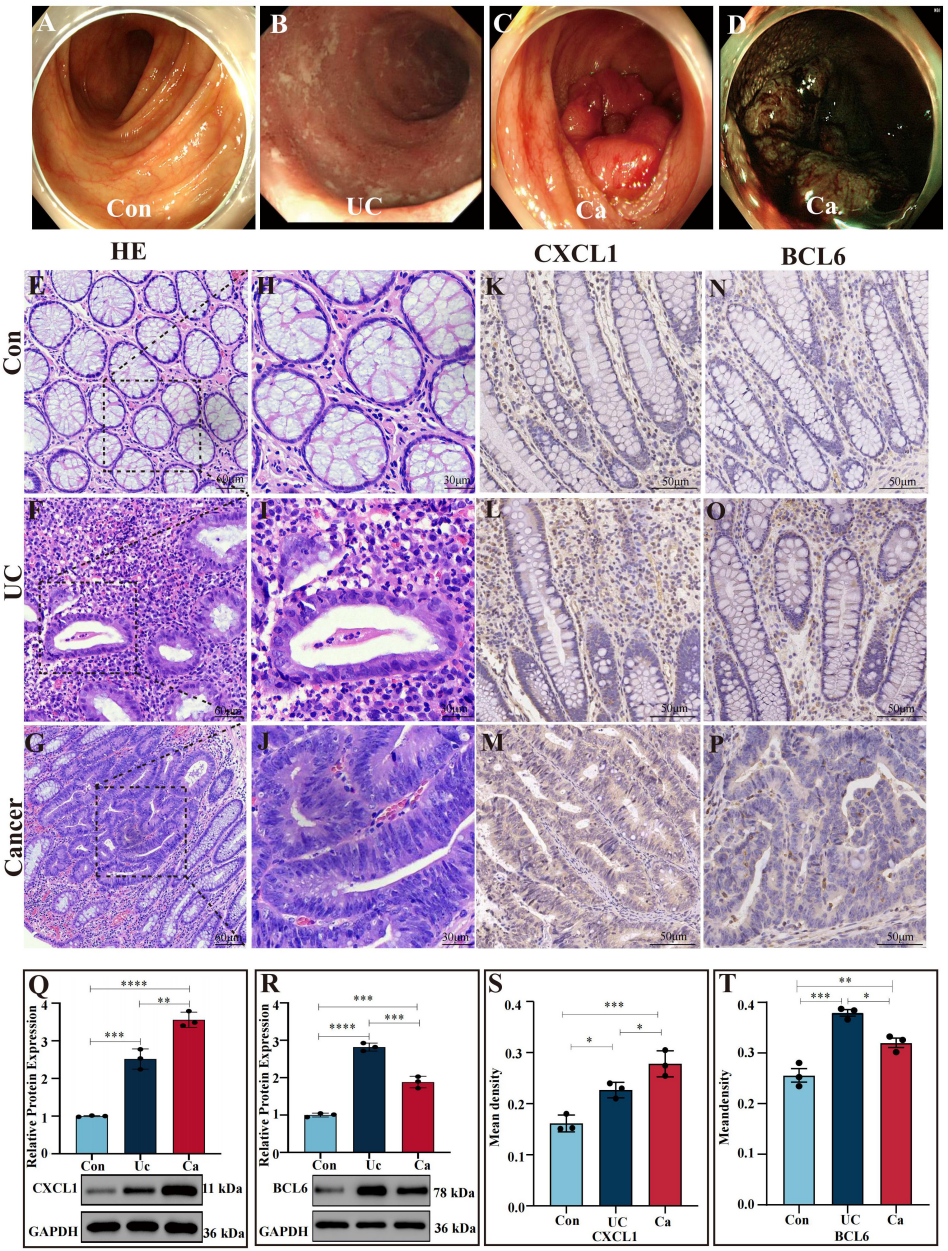
Verification of gene expression in colon tissues **(A)** Images of a normal intestinal tract obtained via white light endoscopy. **(B)** Intestinal tract images of UC patients via white light endoscopy. **(C)** Intestinal tract images of CRC patients via white light endoscopy. **(D)** NBI images of the intestinal tract of CRC patients. **(E, H)** H&E staining of human normal colon sections. **(F, I)** H&E staining of intestinal tissues from UC patients. **(G, J)** H&E staining of intestinal sections from CRC patients. **(K–P)** Expression of CXCL1 **(K–M)** and BCL6 **(N–P)** in the control cohort, UC patient cohort and cancer patient cohort determined by IHC analysis. **(S, T)** Statistical comparison of the IHC analysis of the mean densities of CXCL1 **(S)** and BCL6 **(T)** in the control cohort, UC patient cohort and cancer patient cohort. CXCL1 was expressed at the highest level in the cancer patient cohort. BCL6 was expressed at the highest level in the UC patient cohort. **(Q, R)** Western blot analysis of CXCL1 and BCL6 expression in human intestinal tissues. In the cancer patients group, the CXCL1 expressed the highest than others. In the UC patient group, the BCL6 expression was greater than that in the other groups. *p<0.05, **p<0.01, ***p<0.001 and ****p<0.0001.

Intestinal manifestation of healthy person, ulcerative colitis patients and colon cancer patients under endoscopy (**Figures 6A–D**). Histologic evaluation revealed crypt abscess, mucosal damage and neutrophil infiltration in the intestinal tract of UC patients and mucosal structure disorder and nuclear swelling in CRC patients (**Figures 6E–J**).

### Western blot analysis

Compared to the controls, the UC group expressed more CXCL1 (*P* < 0.001), while the CRC group expressed more CXCL1 (*P*<0.0001), and the CRC group expressed more CXCL1 than UC cohort did (*P*<0.01) (**Figure 6Q**). Moreover, the level of BCL6 in colon tissues demonstrated that the BCL6 in UC patients was markedly greater than CRC patients (*P <*0.001), CRC patients was greater than the control group (*P <*0.001) and in UC patients was greater than the controls (*P*<0.0001) (**Figure 6R**).

### IHC analysis

We applied immunohistochemical staining to determine the mRNA expressions of CXCL1 and BCL6 in nontumor adjacent, UC and cancer tissues (**Figures 6K–P**). CXCL1 in CRC group expressed greater than the UC group (*P*<0.05), CRC group was markedly greater than controls (*P*<0.001), and in UC was greater than the controls (*P*<0.05) (**Figures 6K–M, S**). The levels of BCL6 in UC patients were markedly greater than the CRC patients (*P* < 0.05) and the controls (*P* < 0.001). Moreover, the level of BCL6 in CRC patients was higher than controls (*P* < 0.01) (**Figures 6N–P, T**).

### Protein expression verification of core genes in the intestinal mucosa

**Figure 7:**
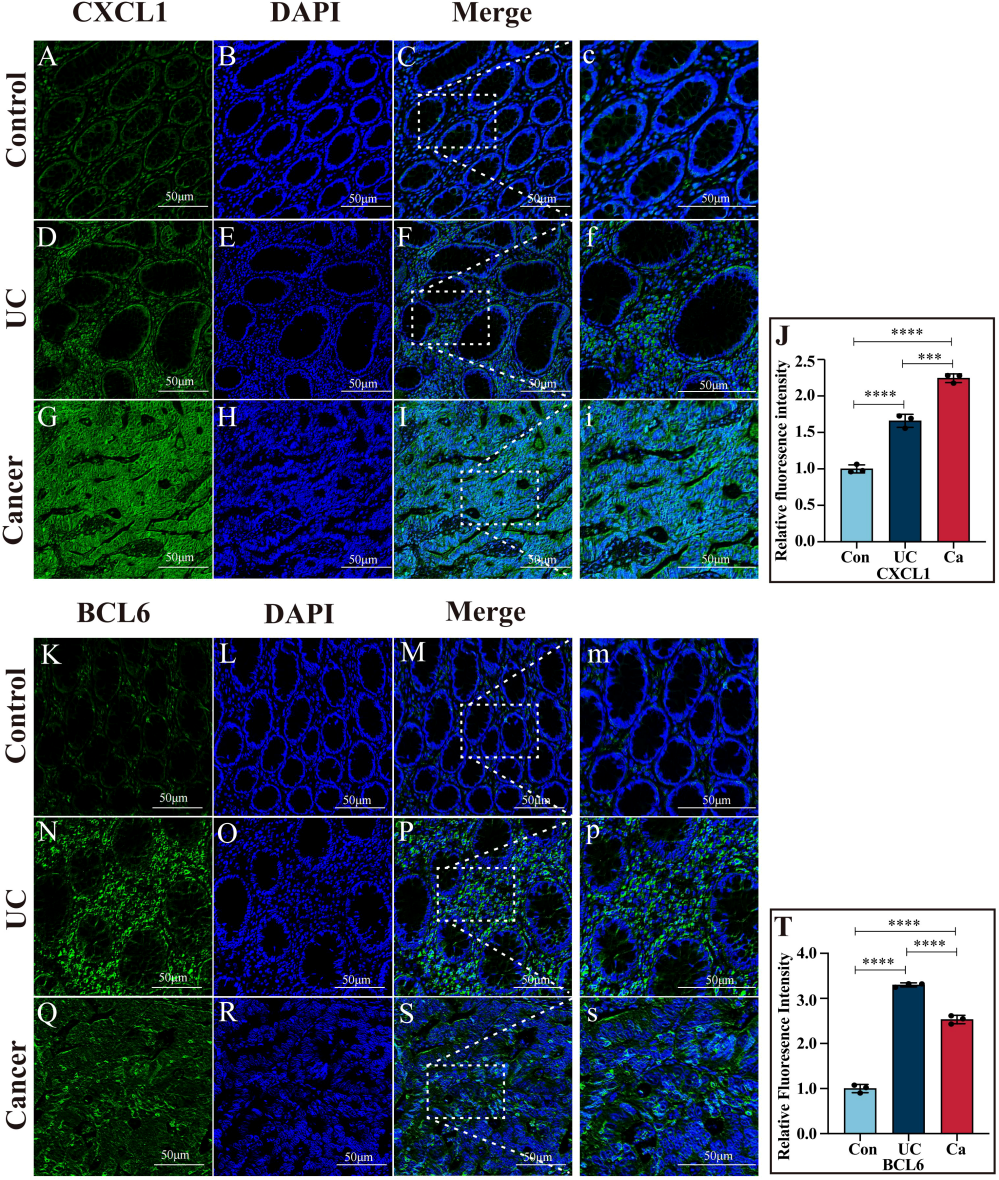
Immunofluorescence images of CXCL1 and BCL6. **(A–I)** Immunofluorescence images of CXCL1 in colon tissues from the control, UC and cancer cohorts. **(J)** Quantitative analysis of CXCL1 immunofluorescence. **(K–S)** Immunofluorescence images of BCL6 in colon tissues from the control, UC and cancer groups. **(T)** Quantitative analysis of BCL6 immunofluorescence. ***p<0.001 and ****p<0.0001.

Immunofluorescence assays demonstrated that, compared with that in the UC cohort (*P*<0.001) and control group (*P*<0.0001), CXCL1 expression in cancer patients was greater and markedly upregulated. The level of CXCL1 in the UC patients was markedly greater than the controls (*P* < 0.0001) (**Figures 7A–J**). The level of BCL6 in the UC patients was greater than the cancer patients (*P* < 0.0001). In contrast with the controls, the level of BCL6 in the UC and cancer patients dramatically increased (*P* < 0.0001) (**Figures 7K–T**).

## Discussion

UCAC often occurs at a more advanced stage in clinical case reports than in other patients with CRC (10). Endoscopies are difficult in the early stages due to the flat UCAC lesion, which leads to increased mortality in UCAC patients (28–30). Thus, we identified two biomarkers (CXCL1 and BCL6) for the diagnosis of colitis-related cancer. The chemokine CXCL1, known as growth-regulated oncogene α, belongs to the CXC chemokine family (31). Chemokines play a vital part in each process of immune system, for instance immune system development and homeostasis (32, 33). Numerous studies have found that tumor cells and tumor microenvironment cells produce chemokines (32). Chemokines can influence the growth of tumor cells, cell survival, angiogenesis, and metastasis (34). Recent extensive research has showed the participation of CXCL1 in various tumorigenesis processes, including lung cancer, melanoma, reproductive cancers, and gastrointestinal cancers (31, 34). Moreover, in many cancers, the expression of CXCL1 may correlate with tumor dimensions, tumor grade, and tumor-node-metastasis (TNM) stage (34). L. Han et al. found a link between CXCL1 and TNM stage in laryngeal squamous cell carcinoma (35). Shi Liu et al. also found a relation between CXCL1 expression and lymph node metastasis in patients with laryngeal squamous cell carcinoma (36). Comprehensive studies on CXCL1’s involvement in colitis-related cancer are relatively rare. This research attempted to address the aforementioned gap by investigating the role of CXCL1 in colitis-related cancer. Studies have demonstrated a connection between the pathogenesis of UC and follicular helper T (Tfh) cells (37). Bcl-6 plays a crucial role in Tfh cell development, as it is essential for its generation (37). According to research by Youguang Yang et al., Bcl-6 changes Tfh/Tfr, promoting the occurrence of IBD (38). However, studies comparing BCL6 and colitis-related cancer are rare. Youguang Yang et al. collected blood samples of IBD patients and detected the level of BCL6 in the normal, UC, and CD groups via PCR. They observed significant increases in BCL6 expression in the ulcerative colitis group (38). In their study, Jiwei Wang et al. found that decreasing BCL6 makes TNF-α-induced apoptosis worse in colonic epithelial cells, which makes UC symptoms worse. Additionally, Wang Team demonstrated that IRF4 exerts a negative regulatory effect on BCL6, facilitating Treg cell differentiation into macrophage-like cells and consequently impeding colon cancer cell proliferation (39). The Jiwei Wang team’s results partially mirrored our own. Our research indicated that BCL6 acts as a protective factor against the progression of CRC, which is consistent with Jiwei Wang’s results. However, our study found that BCL6 expression in the UC cohort was higher than in the controls, which differs from the findings of Jiwei Wang’s team. On the one hand, Jiwei Wang’s team used an acute ulcerative colitis UC mouse model, which contrasts with our long-term tissues from UC patients. On the other hand, there are differences in gene expression between mice and humans. Furthermore, because UC and UCAC have low incidence rates, we included a small sample size.

The NF-kB family is made up of five transcription factors that play a part in immunity, inflammation, cell growth, and the development of cancer (40, 41). NF-kB activation is pivotal in UCAC pathogenesis because it orchestrates the transcriptional upregulation of proinflammatory cytokines, facilitates tumor growth by promoting angiogenesis-related genes, and enhances cell survival via the induction of antiapoptotic genes (41). In mouse models of UCAC, tumor incidence was reduced by ablation of IκB kinase that resulted in NF-κB pathway inactivation (42). In a different study, a UCAC mouse model that had NLRP12 cut out was more likely to get severe colitis and even tumors linked to colitis than wild-type mice. NLRP12 belongs to the Nod-like receptor family and negatively regulates NF-kB signaling (43, 44). These studies further demonstrated that NF-κB plays a significant role in promoting inflammation-driven colon tumorigenesis. In our research, we conducted an enrichment analysis of core genes and found that they were enriched in the NF-κB *p*athways, which indicates that our core genes likely led to the tumorigenesis of UCAC via the NF-κB *p*athway.

**Figure 8:**
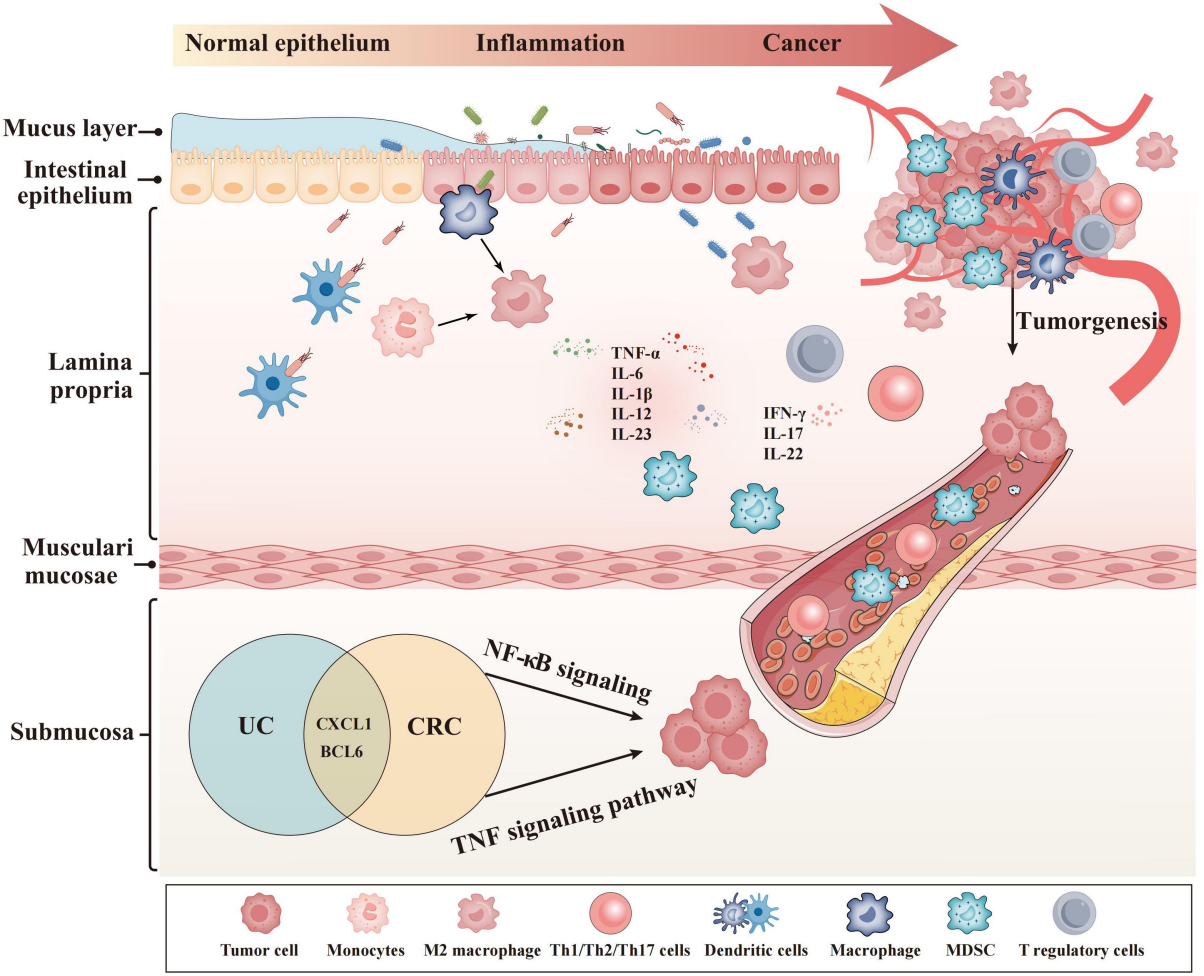
Effects of hub genes on the transformation from ulcerative colitis to colorectal cancer.

The core genes (CXCL1 and BCL6) showed a strong link with macrophages, neutrophils, and B cells in our research. These immune cells were a significant component of the UCAC’s TME (**Figure 8**) (45). The Gao team discovered that TME’s CXC chemokines drive neutrophil production, recruitment, and presence, while also promoting tumor cell survival and extravasation (46). They were consistent with our finding that CXCL1 is positively associated with CRC risk and strongly correlated with neutrophils. Inducing neutrophil polarization toward anti-tumor is then an effective means of improving the CRC prognosis. The Gao team’s results provided direction and a solid foundation for our immunotherapy in the future. In the pan-cancer B-cell Atlas, the Gao team described and investigated the role of BCL6 in the TME of CRC (47). They demonstrated that Tfh played an auxiliary role in B cell proliferation and differentiation and that Bcl-6 was essential for Tfh cell development (47). Given B cells’ role in cancer immunity, we believe that BCL6 could be used for cancer surveillance and treatment in next time.

Using scMetabolism (45), the Gao team demonstrated that macrophages were heavily enriched in CRC and had extremely high metabolic activity. In our research, there was a strong relation between our markers and macrophages in TME. It seems that the core genes suppress the immune system by activating macrophages, which can facilitate CRC development, progression, and even metastasis. Next, we will explore the metabolic landscape of the CRC immune microenvironment using scMetabolism. Meanwhile, the Gao team studied the systemic metabolism and local TME (48). A metabolic competition existed between immune cells and tumors in the TME (48). Metabolism plays an instrumental part in cancer development, and local metabolism intervention represents a promising treatment for CRC.

Although bioinformatic methods predicted the core genes involved in the conversion from UC to CRC, the morbidity of UC and UCAC patients was low, and the sample sizes were small. Therefore, more detailed studies and larger sample sizes are necessary. Multiple factors affected the disease. Thus, advanced comprehensive multiomics studies, such as single-cell sequencing, spatial genomics, and scMetabolism et al., should be performed in our next studies.

## Conclusion

In conclusion, core genes (CXCL1 and BCL6) are effectiveness biomarkers for evaluating the progression from ulcerative colitis to cancer. The level of CXCL1 exhibited a positive relation with UCAC, whereas the level of BCL6 showed a negative relation with UCAC. BCL6 and CXCL1 showed a strong link with macrophages, neutrophils and B cells, which is an important component in TME of UCAC. Our findings provide basic research that targets immune checkpoints to block tumorigenesis and intervene in the heterogeneity of immune cells to play a role in anti-tumor.

## Data Availability

Publicly available datasets were analyzed in this study. The data supporting our study are available in the GEO database, https://www.ncbi.nlm.nih.gov/geo/ and TCGA database https://portal.gdc.cancer.gov/.
