# Supplementary figures and images for: Identification of immune-associated genes for the diagnosis of ulcerative colitis-associated carcinogenesis via integrated bioinformatics analysis
Source: Front Oncol. 2024 Nov 8;14:1475189. doi: 10.3389/fonc.2024.1475189 (PMC11581968; doi:10.3389/fonc.2024.1475189)

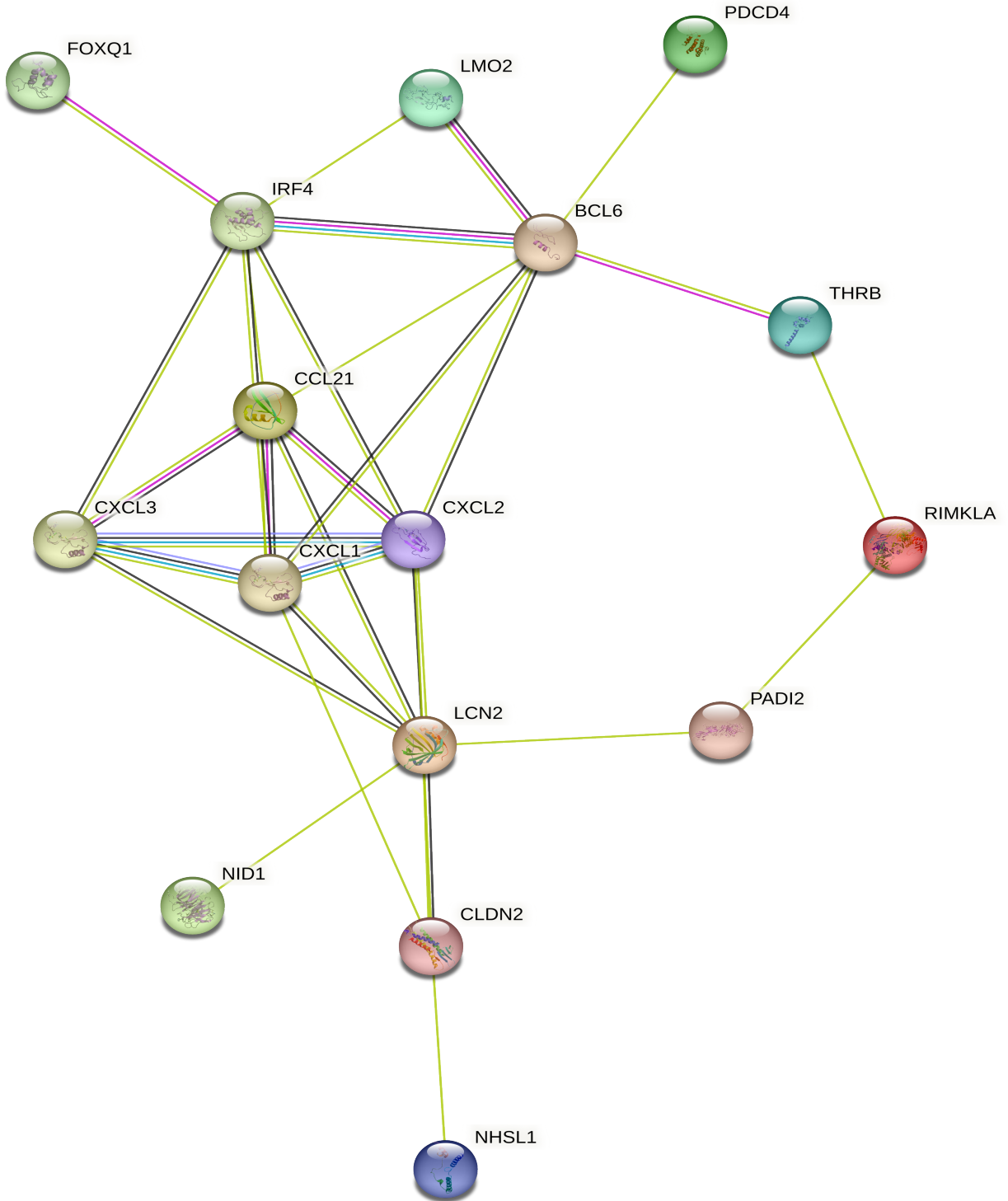

Supplement: Supplementary file 1 [file DataSheet1.pdf]
